# Hepatic Hydatid Cyst Presenting as Right-Sided Heart Failure: A Case Report

**DOI:** 10.7759/cureus.55726

**Published:** 2024-03-07

**Authors:** Taulant Gishto, Leonard Simoni, Mirald Gina, Naltin Shuka, Silvia Methoxha

**Affiliations:** 1 Cardiovascular Disease, University Hospital Center "Mother Teresa", Tirana, ALB

**Keywords:** cyst recurrence, surgical excision, ct scan, hydatid cyst, heart failure

## Abstract

Cystic echinococcosis is a parasitic disease caused by *Echinococcus granulosus*. The transmission of the parasite to dogs occurs when organs of animals that harbor hydatid cysts are consumed.

We present the case of a patient presented in the cardiology outpatient clinic with the signs and symptoms of predominant right-sided heart failure. Upon evaluation, a large hepatic septated cyst was revealed, which was compressing the right chambers of the heart, altering diastolic filling, and causing right-sided heart failure. CT scan confirmed the presence of a hydatid cyst measuring 115 mm × 90 mm. The patient underwent surgical excision of the cyst with immediate relief of the symptoms. Two weeks later, the patient presented again with the same symptoms and was diagnosed with a recurrence of the hydatid cyst. He underwent surgical resection and removal of the cyst again. The patient remained asymptomatic and free of recurrence on further follow-up evaluations.

Cardiac echinococcosis typically features intra-myocardial cysts, while our case presented an extracardiac location. Extrinsic compression of the heart’s right chambers from a hydatid cyst has been rarely reported. The surgical excision of the cyst brings immediate and full resolution of the symptoms. The recurrence of hydatid cysts is also an important clinical feature that should not be underestimated.

## Introduction

Cystic echinococcosis is a parasitic disease caused by *Echinococcus granulosus*. It has a worldwide distribution affecting an estimated 1.2 million people [1,2], mainly in pastoral communities. It is estimated by the 2015 WHO Foodborne Disease Burden Epidemiology Reference Group (FERG) that the number of global annual echinococcosis-related deaths is 19,300, and in addition, this disease also accounts for approximately 871,000 disability-adjusted life years each year worldwide [3].

The transmission of the parasite to dogs occurs when organs of animals that harbor hydatid cysts are consumed; the cysts subsequently mature into adult tapeworms within the canine host. Infected dogs excrete tapeworm eggs in their feces, contaminating the surrounding environment. Livestock, such as sheep, cattle, goats, and pigs, acquire these tapeworm eggs by ingesting the contaminated ground, developing cysts within their internal organs. The most prevalent mode of human infection arises from the accidental ingestion of soil, water, or food contaminated with an infected dog’s feces [4]. It affects the liver in approximately 70% of cases, the lungs in 20% of cases, and other organs in the remaining 10% [5]. Once deposited in soil, the echinococcus eggs can stay viable for a year. Since sheep are intermediate hosts of the parasite, farmers and stockbreeders are most commonly affected [4].

Cystic echinococcosis is an endemic disease in South America, Central Asia, East Africa, and Western Europe, especially Germany, southern France, Turkey, and the Balkan Peninsula [6]. In Albania, the incidence of cystic echinococcosis between 1958 and 1987 was estimated at 2.05 per 100,000 inhabitants [7]. 

The most common clinical signs and symptoms are general malaise and abdominal discomfort, while the most dangerous complication is the rupture of the hydatid cyst, causing anaphylactic shock [4].

## Case presentation

We present the case of a 75-year-old patient who presented to the outpatient cardiology clinic complaining of shortness of breath on minimal exertion and at rest, generalized weakness, fatigue, and palpitations. The patient had high blood pressure under treatment. The patient or his family did not refer to any dog or animal contact history.

On physical examination, a heart with rhythmic sounds, with a systolic murmur audible on the apex; normal vesicular breath sounds, without crackles; and slight to moderate distension of the jugular veins were noticed, and the liver was palpable 4 cm under the costal margin. Blood pressure was 130/80 mmHg, heart rate was 75/min, and respiratory rate was 20/min.

**Figure 1:**
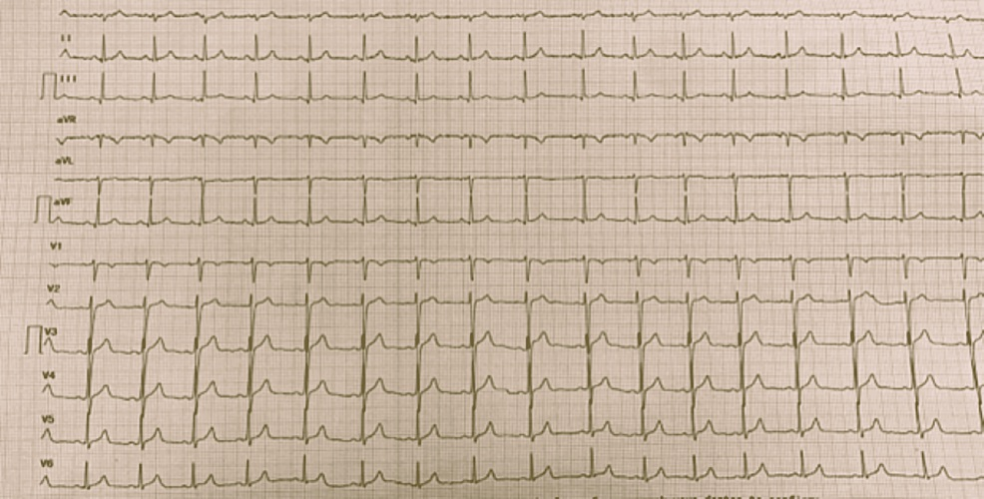
ECG of the patient, without conduction or repolarization abnormalities.

In ECG, sinus rhythm, without conduction disorders, and repolarization abnormalities were found (Figure 1).

Upon admission, her complete blood count and biochemistry panel were within normal range. However, her NTpro BNP level was elevated at 661 pg/mL (normal range < 125 pg/mL) (Table 1).

**Table 1:** Complete blood count and the biochemistry panel. WBC, white blood cells; NEU, neutrophils; LYM, lymphocytes; RBC, red blood cells; HBA, hemoglobin; HCT, hematocrit; PLT, platelets; Creat, creatinine; Na, sodium; K, potassium; Cl, chloride; CRP, C-reactive protein; tot bilirubin, total bilirubin; AST, aspartate aminotransferase; ALT, alanine transaminase; CK, creatine kinase; CK-MB, creatine kinase-myoglobin binding; NTproBNP, N-terminal pro-b-type natriuretic peptide

Complete blood count			
Parameters	Reference range	Units	Patient’s values
RBC	4-5.6	<!--td {border: 1px solid #cccccc;}br {mso-data-placement:same-cell;}--> ×10^6^/uL	4.38
HCT	37-46	%	40.1
HB	12.1-15.9	g/dL	12.8
WBC	4-10.5	K/uL	7.6
PLT	150-400	K/uL	323
Biochemistry panel			
Urea	21-43	mg/dL	28.7
Creatinemia	0.57-1.11	mg/dL	0.63
Na	136-145	mmol/L	142
K	3.5-5.1	mmol/L	3.9
Cl	98-107	mmol/L	101
Tot bilirubin	0.3-1.2	mg/dL	0.76
ALT-SGPT	<55	U/L	15
AST-SGOT	5-34	U/L	29
CK	29-168	U/L	145
CK-MB	<3.1	ng/mL	1.0
Troponin-I	<0.016	ng/mL	0.010
NTproBNP	<125	pg/mL	61.0
CRP	<0.5	mg/dL	0.47
Glucose	82-115	mg/dL	105

**Figure 2:**
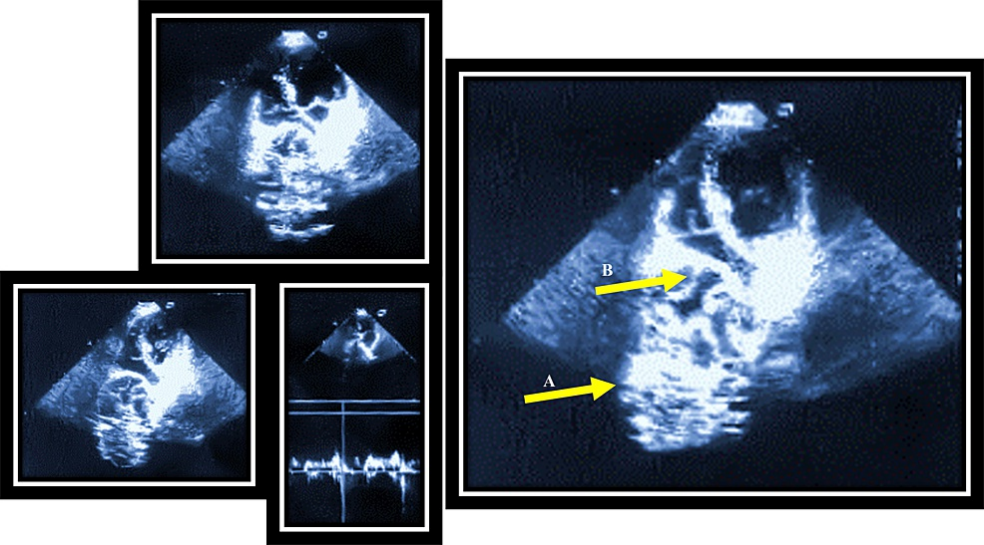
TTE 4-chamber views showing huge extracardiac mass compressing RA and RV, with altered diastolic filling. Arrow A,- extracardiac mass; arrow B, right atrium compression; TTE, transthoracic echocardiography; RA, right atrium; RV, right ventricle

The patient underwent transthoracic echocardiography (TTE) examination, where a multiple-septated cyst formation with a dimension of 120 mm × 87 mm was noticed (Figure 2). The formation seemed capsulated, compressing the heart’s right chambers and causing the complete diastolic collapse of the right atrium (RA) and partial collapse of the right ventricle (RV).

**Figure 3:**
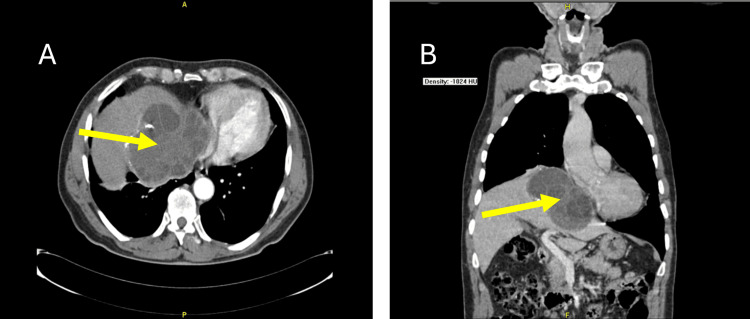
(A) Axial tomographic view at the right atrium level. (B) Coronal tomographic view showing the right chambers collapse from the cystic mass. The arrow shows the cystic mass compressing cardiac right chambers.

The patient was referred for thoracic-abdominal CT examination, which revealed a mass most probably indicative of a hepatic echinococcal cyst with a dimension of 115 mm × 90 mm, elevating the right hemidiaphragm and compressing the right chambers of the heart (Figure 3).

The patient underwent surgical intervention where the encapsulated hepatic cyst was excised. It was confirmed that it was an echinococcus hepatic cyst on pathological examination.

Two weeks after the surgical intervention, the patient presented again to the outpatient cardiology clinic, complaining of a resumption of the previous symptoms. Normal ECG and blood tests were found. A similar, smaller mass was seen on TTE, of less than 50 mm × 40mm and not completely capsulated. It compressed the RA but not the RV. The subsequent CT scan confirmed a recurring echinococcal cyst partially plugged by the omentum, containing 200cc of fluid and warranting surgical revaluation. After that, the patient underwent the surgical resection again one month after the first intervention.

One year after the second surgical intervention, our patient remains asymptomatic without recurrence of the disease according to periodic systematic ultrasound and CT scan examinations.

## Discussion

Hepatic echinococcosis that compresses the right heart chambers is very rarely seen in clinical practice. From our search in PubMed with keywords (hydatid cyst and heart failure), we found two documented cases of right-sided heart failure due to extracardiac compression from a hepatic hydatid cyst. The first one was published in 2000 by Sanchez-Recalde et al., focusing primarily on atrial arrhythmias caused by mechanical extracardiac compression [8]. The second paper was published in June 2009 by Robles et al., presenting right atrial and ventricular compression by a hepatic hydatid cyst [9]. The latter reported probably the largest hydatid cyst ever published in a scientific paper, measuring 155 mm × 115 mm [9]. In our case, the cyst measured 115 mm × 90 mm, which, to the best of our knowledge, appears to be the second largest cyst presenting with right-sided heart failure.

The compression of the heart’s right chambers from extracardiac masses is not usually encountered in clinical practice. The cardiac compressions are observed from various mediastinal or intrapericardial tumors or cysts, dilated descending aorta, and hiatal hernia [10]. The cysts usually are intramyocardial of the left ventricle in cardiac echinococcosis [11]. It is worth mentioning that dyspnea, which was the clinical symptom presented by the patient, can even be caused by extracardiac masses compressing the right heart chambers and producing this clinical situation. The surgical excision of the cyst produces immediate and full resolution of the symptoms.

Despite the therapies now available, in various series, hydatid cyst recurrence remains at a higher range from 4.6% to 22.0% [12-14]. According to the WHO, about 6.5% of cases relapse after an intervention [3]. In the series of Prousalidis et al., the recurrence rate was 8.7%, and the two most important determinants for recurrence were minute spillage of the hydatid cyst and inadequate treatment owing to missing cysts or incomplete pericystectomy [12]. In the series of Mottaghian and Saidi, the recurrence rate was 11.3%, with no correlation found between the size of the removed cysts and postoperative recurrence [13]. Kapan et al. concluded that proper incision selection allows complete exposition, and the performance of pericystectomy in solitary, peripherally located cysts prevents recurrence. In their series, the recurrence rate was 4.65% [14].

## Conclusions

Extrinsic compression of the heart’s right chambers from a hydatid cyst has been rarely reported. Cardiac echinococcosis typically features intra-myocardial cysts, while our case presented an extracardiac location. Dyspnea, which was the main clinical symptom presented by our patient, can be caused by other extracardiac masses compressing the right heart chambers and producing this clinical situation; therefore, a differential diagnosis must be made. The surgical excision of the cyst brings immediate and full resolution of the symptoms. The recurrence of hydatid cysts is also an important clinical feature that should not be underestimated.
